# 质谱数据处理软件XCMS在环境科学领域的应用综述与研究展望

**DOI:** 10.3724/SP.J.1123.2025.01019

**Published:** 2025-06-08

**Authors:** Cheng YANG, Ao ZHANG, Zhanqi GAO, Guanyong SU

**Affiliations:** 1.南京理工大学环境与生物工程学院，江苏省化工污染控制与资源化高校重点实验室，江苏 南京 210094; 1. Jiangsu Province Key Laboratory of Chemical Pollution Control and Resources Reuse，School of Environmental and Biological Engineering，Nanjing University of Science and Technology，Nanjing 210094，China; 2.江苏省环境监测中心，生态环境部地表水环境有机污染物监测分析重点实验室，江苏 南京 210019; 2. Key Laboratory of Environment Monitoring and Analysis for Organic Pollutants in Surface Water，Ministry of Ecology and Environment，Jiangsu Province Environmental Monitoring Center，Nanjing 210019，China

**Keywords:** XCMS, 环境科学, 非靶向筛查, 未知污染物, XCMS, environmental science, non-targeted screening, unknown contaminants

## Abstract

生物样品和环境样品中化合物种类繁多、成分复杂，使用色谱-高分辨质谱对样品进行分析后会产生大量由质荷比（mass-to-charge ratios，*m/z*）、保留时间（retention-time，RT）、峰强度等组成的色谱-质谱数据，处理这些数据需要耗费大量的时间和精力，需要借助质谱数据处理软件对其进行识别分析。在众多的质谱数据处理软件中，各种形式的色谱质谱（various forms （X） of chromatography mass spectrometry， XCMS）作为一款高效、准确且可免费获取的质谱数据处理软件，在环境科学领域得到广泛应用。本论文聚焦XCMS在环境科学领域中的应用，综述了XCMS的工作流程、工作原理和参数优化措施。XCMS的工作流程主要包括数据导入、数据处理和数据导出等步骤，数据导入需要借助MSConvert等格式转换工具将不同仪器生成的数据转换为XCMS可接受的格式，数据处理大致包括峰检测、峰对齐和峰填充等步骤。在应用方面，XCMS在环境污染物非靶向筛查、污染物外源性代谢转化鉴定以及生物分子内源性代谢研究中取得了显著进展。例如，在环境污染物非靶向筛查中，XCMS能够高效提取复杂样品中的质谱特征，为后续的鉴别提供可靠的数据基础。尽管XCMS在环境科学领域的应用取得了一定成效，但仍存在一些局限性，如用户交互和自动化程度仍有待提高。XCMS在环境科学领域的发展潜力巨大，未来随着算法的不断优化和数据库的扩展，通过不断改进算法鲁棒性、数据兼容性和用户体验，XCMS有望为环境科学研究提供更强大的支持。

## Abstract (en)

Biological and environmental samples are complex and contain a highly diverse range of compounds. Analyzing these samples by chromatography-high-resolution mass spectrometry generates a substantial volume of mass-spectrometry data that are composed of mass-to-charge-ratio （*m/z*）， retention-time （RT）， and peak-intensity information that require considerable time and energy to process. Consequently， employing software to process mass-spectrometry data for identification and analysis purposes is imperative. Among the many mass-spectrometry data-processing options， XCMS （various forms （X） of chromatography mass spectrometry）， which is highly efficient， precise， and freely accessible software for processing mass-spectrometry data， is broadly used in the environmental science field. This study aimed to explore the use of XCMS in environmental science applications by comprehensively reviewing the workflow， underlying principles， and parameter-optimization measures of XCMS. The workflow mainly includes importing， processing， and exporting data. Importing data requires the use of format conversion tools， such as MSConvert， which converts data generated by various instruments into a format acceptable by XCMS， while data processing includes peak detection， alignment， and filling. The various XCMS functions are mainly realized via its built-in algorithms， with the Matched Filter， CentWave， Obiwarp， and Peak Density algorithms most commonly used. The first two algorithms implement the peak-detection function， while the latter two implement the peak-alignment function. XCMS identifies compound peaks from mass-spectrometry data during peak-detection； it first filters for noise and corrects the baseline. An algorithm then detects peaks based on their shapes and intensities. XCMS can also de-emphasize and de-distort to filter out interfering information in each peak signal. The CentWave algorithm is particularly effective for processing high-resolution mass-spectrometry data by improving detection accuracy and recall. Peak-detection is followed by alignment. Here， XCMS uses kernel density estimations to match peaks between samples by estimating the retention-time distribution of matched peaks， which corrects for any nonlinear deviations in retention-times. This step is critical for accurately comparing samples. The peak-filling step resolves missing peaks in the data， and XCMS uses information from other samples to fill these gaps. This process enhances the integrity of the dataset and improves analysis accuracy. In terms of applications， XCMS has demonstrated significant progress for the non-targeted screening of environmental pollutants， identifying exogenous metabolic pollutant transformations， and exploring the endogenous metabolisms of biomolecules. For example， XCMS efficiently extracts the mass spectrometry of complex samples during the non-targeted screening of environmental pollutants， thereby providing a reliable database for subsequent identification. Although the use of XCMS in the environmental science field has delivered particular results， some limitations still exist， including the use of large amounts of memory， problems associated with the software crashing when dealing with large-scale data， and the misclassification of noise as valid signals during feature detection， which results in a large number of false positives， errors， and missed detections when processing data for compounds with complex chemical compositions and structural types. In addition， the degree of user interaction and automation requires further improvement. XCMS offers significant developmental potential in the environmental science field. Continuing algorithmic optimization and database expansion through improvements in algorithmic robustness， data compatibility， and user experience， are expected to see XCMS develop broadly and provide more powerful support for the environmental science field in the future.

随着高分辨质谱技术的快速发展，环境样品和生物样品中的复杂化合物分析变得越来越普遍，这些分析产生的海量质谱数据需要借助专业的质谱数据处理软件进行解析。目前常用的质谱数据处理软件包括商业软件和开源工具两大类。商业软件具有图形界面友好、自动化程度高的特点，但通常价格昂贵且灵活性较低，如Compound Discoverer、Progenesis QI等。开源工具如MZmine、MS-DIAL和各种形式的色谱质谱（various forms （X） of chromatography mass spectrometry， XCMS）等，因其免费、灵活且可定制化强，逐渐成为研究人员的重要选择。

在众多开源工具中，XCMS因具有高效、准确且免费的特点，在环境科学领域得到了广泛应用。XCMS是一款由美国加州斯克利普斯研究所（Scripps Research Institute）的Gary Siuzdak教授领导的代谢组学研究团队开发的软件工具，其目的是提高质谱数据分析的效率和准确性^［1］^。该软件采用R编程语言编写，并通过Bioconductor平台以通用公共许可证（GPL）或GNU开源许可方式发布，支持在多种操作系统上运行，包括UNIX、苹果的OS X以及Microsoft Windows系统^［2］^。

自2006年首次发布以来，XCMS凭借其优秀的数据处理能力和广泛的适用性，受到代谢组学等领域研究人员的广泛关注，常被应用于基于液相色谱-质谱（liquid chromatography-mass spectrometry， LC-MS）代谢组学数据分析。原始的XCMS软件使用Matched Filter算法来完成特征检测，使用retcor.peakgroups算法来执行比对，并且使用group.density算法基于*m/z*区间对样品之间的比对特征进行分组^［1］^。随着质谱技术的快速发展以及检测样品复杂性的增加，研究人员开发了更为准确的CentWave新特征检测算法^［3］^和Obiwarp（ordered bijective interpolated warping）新对齐算法^［4］^，大大提升了XCMS的数据处理性能和准确度。目前，XCMS数据处理算法还在不断改进升级，以满足日益增长的数据处理需求。

除了XCMS单机版本外，Gary Siuzdak教授领导的代谢组学研究小组还在2012年推出了XCMS线上版本，即XCMS Online^［5］^。该版本不仅保留了原始XCMS软件的相同功能，还新增了统计分析功能。用户无需熟悉命令行界面或编程技能，只需上传LC-MS仪器采集获取的数据，调整相关参数，即可获得所需结果。然而，与原始的XCMS相比，XCMS Online提供的资源和配置较为固定，不支持自定义硬件、系统配置或安装自定义插件，因此灵活性较低，研究人员可根据自身需求选择合适的版本。Jurich等^［6］^比较了MVAPACK、XCMS Online和MS-DIAL这3种质谱数据处理软件性能，XCMS Online识别了模拟数据集中935个具有统计学意义的代谢特征中的735个，在3个软件中表现最佳。表1列举了XCMS和XCMS Online的优缺点。

**表 1:** XCMS与XCMS Online的优缺点对比

Platform	Introduction	Advantages	Disadvantages	Ref.
XCMS	processing LC-MS raw data based on R packages	customizable parameters for complex analysis scenarios	requires knowledge of R language	［^1^］
XCMS Online	web-based graphical user interface version of XCMS	data can be stored and shared in the cloud； graphical interface， easy to use， no programming foundation	simplified parameter settings for standardized processes， but less flexible	［^5^］

基于文献的检索结果，除了生命科学领域外，XCMS也越来越多地被应用于环境科学领域，大大促进了环境污染物的非靶向筛查^［7］^、环境样品的非靶向代谢物分析^［8］^、环境中污染物转化过程研究^［9］^以及环境中污染物暴露对生物代谢影响分析^［10］^等方面的发展。本论文聚焦XCMS在环境科学领域的应用，总结了XCMS的工作原理和在环境污染物研究过程中的应用实例，进而展望了XCMS的应用局限以及未来发展方向。

## 1 XCMS的工作流程、原理以及参数优化

### 1.1 XCMS的工作流程

液相/气相色谱-质谱的原始数据是由质荷比（mass-to-charge ratios，*m/z*）、保留时间（retention-time，RT）和峰强度组成的一个三维数据集。数据预处理是为了准确提取各物质的质谱信息，生成一个峰强度与其对应的*m/z*和RT的二维峰强度列表，XCMS对质谱数据处理主要包括峰检测、峰对齐以及峰填充这3个步骤。图1显示了XCMS的一般工作流程。

**图1:**
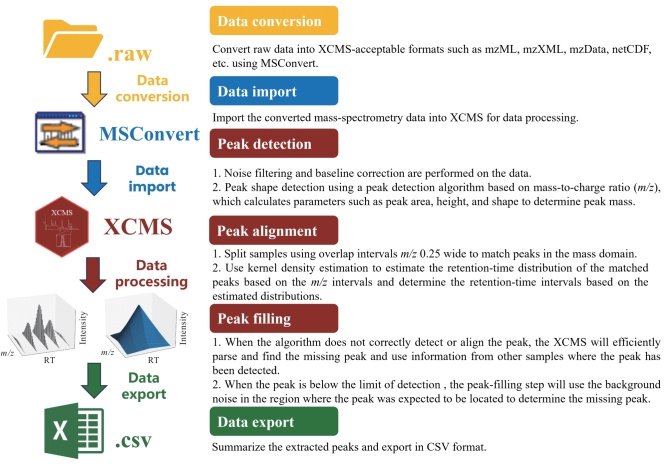
XCMS的一般工作流程

#### 1.1.1 数据导入

由于每个仪器供应商使用的数据格式不同，在用户将所需的质谱数据导入XCMS处理之前，需要利用格式转换工具，例如MSConvert，将数据格式转换成XCMS可接受的格式。目前，XCMS支持多种常见格式，包括mzML、mzXML、mzData、netCDF等，用户可以根据需要选择合适的格式导入^［11］^。

#### 1.1.2 峰检测

峰检测是XCMS中的一个重要步骤，可以识别质谱数据中的化合物峰。在峰检测之前，XCMS会对数据进行噪声过滤和基线校正，便于峰值检测并且潜在地减少了假阳性特征的检测。接着采用一种基于*m/z*的峰检测算法，先对数据进行峰形检测，然后通过计算峰的面积、高度、形状等参数来确定峰的质量。在峰形检测中，XCMS采用一种基于高斯分布的算法，将峰形拟合为高斯分布曲线，然后根据曲线拟合参数来确定峰的位置和形状。在峰形检测后，XCMS还可以进行去重和去畸变处理，以过滤峰信号中的干扰信息^［1］^。

#### 1.1.3 峰对齐

XCMS使用*m/z* 0.25宽的重叠区间拆分样品，以匹配质量域中的峰，然后基于*m/z*区间使用核密度估计法估计匹配峰的保留时间分布，并基于估计的分布确定保留时间间隔。如果在过程后出现多个匹配峰，则使用最接近中位保留时间的峰断开连接。此外，XCMS具有可选的保留时间对齐功能，该功能使用一组“表现良好”的峰（well-behaved peak groups， WBPG）作为临时标准，计算每个样品保留时间的非线性偏差并进行校正^［9］^。

#### 1.1.4 峰填充

在峰检测和对齐的过程中，可能导致峰被遗漏的主要原因有两个：一是算法未能正确检测或对齐该峰，二是该峰的强度低于检出限，导致在峰对齐后显示为零，从而影响分析结果的准确性。当峰被遗漏是由算法导致的，即数据中存在峰，但未被正确检测或对齐，XCMS会利用在其他样品中检测到的相应峰的信息，如保留时间和*m/z*进行峰填充。在第二种情况下，当峰的强度低于检出限时，峰填充步骤将使用预期峰所在区域中的背景噪声来估算缺失的峰值^［3］^。

### 1.2 XCMS的工作原理

XCMS的各种功能主要通过其内置算法来实现，下面将详细介绍XCMS中常见的4种算法，分别是Matched Filter算法、CentWave算法、Obiwarp算法及Peak Density算法，前两种算法实现了峰检测功能，后两种实现了峰对齐功能。

#### 1.2.1 Matched Filter算法

Matched Filter算法为XCMS的初始峰检测算法，其主要流程如下：首先把数据分割成*m/z* 0.1宽的片段，然后在色谱时域中对这些单独的片段进行操作。在每个片段内，算法会检测信噪比高于临界值（默认值为10）的峰。峰检测是利用色谱峰的典型类高斯形状来执行的，这意味着如果这些数据点能很好地拟合为二阶导数高斯分布曲线（基本上是正态分布），则它们被检测为峰。此高斯曲线的宽度由半峰全宽参数定义。该参数是以秒为单位的值，此参数的值越低，越可能检测到假阳性峰（检测为峰的噪声）。为了确保峰不会在两个*m/z*区间之间分裂，该算法将连续*m/z*区间对合并。Matched Filter算法主要针对低分辨率仪器（如单四极杆质谱仪）采集的数据，其最高质量准确度约为0.1 Da，而对于高分辨率质谱仪，通常使用CentWave算法^［1］^

#### 1.2.2 CentWave算法

近年来，研究人员开发了一种新型的峰检测算法——CentWave，与原始的峰检测算法Matched Filter相比，它具有更高的整体召回率和准确率。该算法包括两个步骤：第一步是进行动态分箱，即寻找包含峰的潜在区域，列为感兴趣区域；第二步是使用高灵敏度小波滤波器在这些潜在区域内进行峰值检测。该算法寻找连续扫描中*m/z*偏差较小且信号强度持续高于噪声水平的区域作为感兴趣区域。控制该行为的主要参数是“百万分之一”（parts per million， ppm），该参数选择感兴趣区域的*m/z*跨度和峰宽，峰宽以秒为单位测量，并指示色谱时间中的峰长。一旦发现满足这些要求的感兴趣区域，会通过小波滤波器对其进行分析。使用的小波是墨西哥帽小波，其模拟峰形允许在该感兴趣区域内选择多个紧密洗脱的峰。调整峰值的比例或高度，直至最佳拟合。如果不满足拟合参数，则感兴趣区域会被拒绝作为峰值^［3］^。

#### 1.2.3 Obiwarp算法

Obiwarp算法是XCMS中一种峰对齐算法。该算法将每个样本峰值分成多个片段，并计算每个片段的质量。接着，选取一个样本作为参考样本，并将其峰值映射到参考时间轴上。计算参考样本中每个片段的质量，并标记那些质量较低的片段。然后，利用参考样本中的峰值信息，将每个样本的峰值映射到参考时间轴上。根据参考样本中标记的低质量片段，去除那些映射到低质量片段的峰值。此后，利用所有样本的映射信息，计算一个峰值的平均位置，作为该峰值在参考时间轴上的位置。最后，通过非线性插值，将每个样本中的峰值插值到参考时间轴上，从而完成时间对齐。该算法可以有效减少峰值偏移，提高质谱数据的准确性和可靠性^［4］^。

#### 1.2.4 Peak Density算法

Peak Density算法是XCMS中的另一种峰对齐算法。该算法通过查找样品中聚集在某个保留时间周围的峰组来发现WBPG，这些峰组随后将用于对齐色谱的其余部分，从而提高总体峰分组的准确性。为了找到这些WBPG，该算法使用核密度过滤器来精确定位具有WBPG的区域。可通过调整带宽（bandwidth，bw）参数控制滤波器，对于高效液相色谱数据，常见的默认值是30 s。WBPG需要在保留时间内分散，从而进行良好的非线性校正。使用每个WBPG的中位保留时间，可以通过局部估计散点图平滑的回归技术获得比对曲线。此技术是一种非参数技术，它通过将每个色谱峰拟合成一条平滑线，比较色谱峰间的这些平滑线，从而最终校正样品间的保留时间^［12］^。

### 1.3 XCMS处理参数及优化措施

XCMS对质谱数据进行处理时，处理参数（峰宽、保留时间、背景噪声等）数据必须与和实验设备相匹配，以便在保持低假阳性率的同时，优化真实质谱峰的数量^［13］^。目前研究人员已开发出一些XCMS处理参数优化策略。Libiseller等^［14］^开发了针对XCMS执行非靶向筛查参数优化的软件Isotopologue Parameter Optimization （IPO）。该软件基于样品中天然同位素^13^C和^12^C相对丰度的比例关系构建优化方程，使用梯度下降法对最小峰宽、最大峰宽等XCMS非靶向数据处理过程中的参数进行优化，首先进行参数范围初筛，利用Box-Behnken设计等统计学方法生成少量但有代表性的参数组合，模拟“多组对照实验”运行测试，每组参数运行后，通过响应面分析等数学模型评估结果质量（如峰检测准确率等），锁定表现最佳的区域并动态调整参数范围，经过这样自动评估与迭代，逐步逼近最佳参数，提高了146%~361%的真阳性结果，将假阳性结果减少了3%~8%。Albóniga等^［15］^对猪肝脏和血浆样本的高效液相色谱-飞行时间质谱代谢组学数据进行了研究，比较了IPO自动优化XCMS参数和手动优化参数的效果，结果表明IPO自动优化法在信号强度高、色谱峰宽合理、数据重复性好的肝脏样本中检测的特征数量多（5 603个），在信噪比低、重复性差的血浆样本中手动优化虽然耗时，但更能确保数据的可靠性。McLean等^［16］^运用建立的机器学习算法，利用梯度下降法对XCMS在数据处理过程中的参数持续改进，最终确定了最优的非靶向数据处理参数组合。这一方法有效减少了假阳性结果的出现。Sadia等^［17］^针对饮用水样本中全氟和多氟烷基物质（PFAS）的非靶向检测算法优化进行了研究，发现XCMS通过参数优化（如降低信噪比）显著提升了特征检测数量（从19 652增至21 859），同时将假阳性率降低12%，验证了XCMS在平衡检测灵敏度与计算效率方面的关键作用。

## 2 XCMS在环境科学领域中的应用

### 2.1 环境中污染物筛查

得益于仪器技术的快速进步，非靶向筛查技术在环境污染物的识别分析中得到了广泛应用。该技术依赖于色谱-高分辨质谱仪、核磁共振等分析仪器对环境样品进行质谱数据的采集，借用质谱数据处理软件（XCMS、MZmine等）对所采集的信息进行解析，进而利用商业化或自建图谱数据库、碎片预测等途径获得样品中的化合物信息^［18］^。XCMS是目前在非靶向筛查中应用最为广泛的数据处理软件之一，本节主要介绍XCMS在环境污染物非靶向筛查和代谢转化鉴别中的应用。

#### 2.1.1 环境污染物非靶向筛查

环境样品中包含的化合物种类繁多、结构各异，利用色谱-质谱技术对样品进行检测后会产生大量质谱数据，而非靶向筛查的难点之一就是将这些数据可视化并进行系统的分析^［19］^。XCMS可以快速地对质谱数据中的峰进行识别、过滤、对齐、提取，并扣除背景中的信号峰，将筛选出的有机分子特征汇总到工作表中，研究人员再根据这一列表结合相关软件完成进一步的分析和鉴定。Szabo等^［20］^对回收纺织品中新污染物进行检测与分析，使用XCMS实现了对样本中质谱特征的高效提取与对齐。在正离子模式下，XCMS从所有样本和空白中提取并对齐了114 965个特征，经初始阈值过滤和峰质量评估后，保留了14%的特征（16 027个），其中5 999个特征具有MS^2^谱图；负离子模式下，提取的53 068个特征中，8%（4 092个）被保留，1 417个具有MS^2^谱图。这一结果表明，XCMS显著提升了复杂质谱数据的处理效率，为后续非靶向筛查及化学物质优先级评分提供了可靠的数据基础，最终实现了6种欧盟管控的持久性、迁移性和毒性物质（PMT）及43种纺织相关化学物质的识别与风险评估。

然而，利用高分辨质谱对复杂环境样品进行分析会产生数以万计的色谱峰，使用非靶向识别策略解析所有检测到的质谱峰需要消耗大量的时间和精力。因此，对XCMS检测到的质谱峰进行筛选，选择感兴趣的峰显得尤为重要。Alygizakis等^［7］^连续8天从雅典污水处理厂采集24 h复合流比例进水样品，使用液相色谱-四极杆飞行时间质谱对样品进行检测，接着利用XCMS对质谱数据进行峰提取、峰匹配和保留时间对齐，然后使用贝叶斯方法对输出的化合物列表进行统计检验，找出每日样品中高波动的化合物，对其进行优先级排序。结合高分辨质谱数据库、化学数据库、计算机模拟碎裂工具和保留时间预测模型初步鉴定了14种污染物，其中有两种污染物首次在废水中被检测。色谱峰的识别与提取是非靶向筛查流程中的重要一环，XCMS凭借其强大的算法功能为研究人员提供了较为全面的色谱峰信息，极大地提升了环境污染物非靶向识别的效率与准确性。

#### 2.1.2 外源性污染物转化研究

XCMS最初为处理非靶向代谢组学数据而开发，作为开源软件具有免费获取、操作便捷等特点，精准的峰检测与提取功能使该软件迅速获得广泛应用，并被用于识别新污染物的转化产物。在使用过程中，研究人员首先会对污染物的转化途径进行预测，常见的转化途径预测方法有实验模拟、计算模型预测、数据库匹配、机器学习预测等，接着利用特征离子筛选XCMS检测到的质谱峰，得到疑似目标离子后再进行进一步分析鉴定。钟蔚等^［21］^以含有消毒副产物的河北省各地地下水为研究对象，采用Dionex UltiMate 3000与Q-Exactive Focus联用系统对样品进行分析，借助XCMS的CentWave和Peak Density算法对原始数据进行峰提取与峰匹配，同时基于XCMS提供的chromPeak Spectra函数提取二级质谱，将二级质谱和峰列表转换为本地关系数据库。通过SQL脚本筛选二级质谱中存在碘离子碎片的离子，获取疑似碘化消毒副产物，再通过分子式计算和结构解析进行鉴定，最终鉴定出大量结构不同且毒性较强的碘化消毒副产物。Rocha等^［22］^在研究牛血清中庚酸睾酮滥用的监测策略时发现，XCMS结合MetaboAnalyst平台对非靶向代谢组学数据处理具有关键作用：通过优化参数（信噪比≥5等），可以从非靶向代谢组学数据的10 447个特征中筛选出3个关键生物标志物，该方法不仅表现出较传统靶向方法更优异的灵敏性和特异性，还将检测窗口扩展至92天，表明XCMS在复杂生物标志物的发现及高通量监测中的技术优势。

当研究污染物与某一特定化合物结合后的转化产物时，研究人员可借助XCMS Online新增的统计分析功能进行数据处理，这为研究人员省去了一些手动分析的步骤，提高了研究效率。Segura等^［9］^采用超高效液相色谱与线性离子阱-轨道阱质谱联用系统分析了含低剂量臭氧的左氧氟沙星溶液和不含臭氧的左氧氟沙星溶液，利用XCMS Online差示分析比较两组样品，鉴定显著增加或减少的峰。根据获得的峰列表进行分子式预测，在超高效液相色谱与四极杆飞行时间质谱联用系统中通过串联质谱实验对潜在的臭氧转化产物进行结构解析，成功鉴定出左氟沙星经臭氧分解后的左氟沙星氮氧化物。

### 2.2 内源性生物小分子非靶向识别与代谢影响研究

代谢组学是一种新兴的研究生物学机制的方法，它通过对生物体、组织或细胞中的代谢物进行非靶向综合分析，确定生物体系受到外界因素干预后所产生的各种代谢物的质和量及其变化规律^［23］^。本节主要介绍XCMS在非靶向识别生物小分子以及污染物暴露对生物体代谢影响中的具体应用。

#### 2.2.1 内源性生物小分子非靶向识别

通过液相色谱-质谱法对环境样品中的代谢物进行全局分析，会产生数据集过大而无法手动评估的问题^［24］^。幸运的是，现在有各种各样的软件程序可用于自动化数据分析。非靶向液相色谱-质谱数据同时包含了环境污染物和内源性代谢物的信息，研究人员需要借助质谱数据处理软件进行分析，不同的化合物选用的数据库不相同，如内源性代谢物的代表性数据库有人类代谢组学数据库（HMDB）、KEGG数据库等，环境污染物的代表性数据库有PubChem、EPA DSSTox等。XCMS是目前应用较为广泛的软件之一，它帮助研究人员从大量且复杂的质谱数据中提取代谢物的相关特征，然后研究人员可以基于获得的特征结合相关软件以及代谢数据库完成代谢物的鉴定。利用XCMS对农作物代谢产物进行鉴定，已成为市场上不同农作物产地溯源的有利依据，具有广泛的实用性和经济价值。苗玥等^［25］^采用超高效液相色谱-质谱系统对云南小粒咖啡生豆和埃塞俄比亚咖啡生豆进行分析，利用XCMS对质谱数据进行峰识别、提取、对齐、积分等处理，然后与BiotreeDB 2.1自建的二级质谱数据库进行匹配分析，接着利用SIMCA 14.1软件对代谢组学结果进行单变量和多变量分析，从而找出组间差异代谢物。最终筛选出36种可以区分不同产区咖啡豆的差异代谢物。

此外，利用XCMS分析鉴定植物代谢产物，有助于揭示该植物的生理与代谢适应机制，并为其科学开发和质量控制提供参考。王纪阳等^［26］^利用Ultimate 3000超高效液相色谱与四极杆静电场轨道阱线性离子阱杂合型质谱联用系统对在新疆阿勒泰富蕴地区采集的不同生长时期的一枝蒿植物样本进行分析，利用XCMS软件包对质谱数据进行峰识别、峰对齐、峰填充及峰过滤等操作，之后采用SIMCA 15.0软件构建幼苗期与盛花期样本的模式识别模型，通过*t*检验和Pearson相关性分析等步骤筛选出可靠离子，基于代谢物数据库HMDB检索和已知成分的质谱裂解规律，推测代谢物的结构或结构类别。最终获得了与该植物生长发育密切相关的24种代谢物，为新疆一枝蒿的质量控制和合理利用提供了指导。XCMS的应用极大地提高了非靶向代谢组学数据处理的效率，随着其内置算法功能的不断加强，未来在非靶向代谢组学领域将有更大的应用空间。

#### 2.2.2 环境污染物对生物小分子代谢的影响

目前，非靶向代谢组学在探究环境暴露对生物体的潜在毒性影响方面正受到越来越多的关注。通过评估暴露引起的内源性代谢变化，可以确定受影响的途径和作用机制^［27］^。在该过程中，XCMS用于提取暴露前和暴露后的生物样品中的代谢物特征，研究人员再结合相关软件分析暴露前后样本之间的代谢物差异以及暴露对代谢物组成、数量和通路等方面的影响。需要注意的是，在利用XCMS进行代谢研究时，主要是将其作为质谱数据特征发现工具，其本身不具有自动区分暴露物和代谢物的能力，通过实验设计优化、数据库匹配、标准品和多组学验证等方式可有效排除暴露物的干扰。Li等^［28］^以接触苯的工人和苯中毒小鼠为研究对象，利用XCMS软件对液相色谱-质谱数据进行了峰对齐、保留时间矫正及峰面积提取，评估了长期接触苯对健康的影响。结果表明长期接触苯的工人会出现血糖水平降低以及白细胞计数和尿酮体的变化，血浆中能量和脂质代谢紊乱。长期接触苯的小鼠会有器官受损、体重减轻、氧化应激及嘌呤和脂质代谢异常。张梦妍等^［29］^以成年斑马鱼为研究对象，将其暴露于不同浓度的4-氯-3-甲基苯酚中，采用超高效液相色谱-三重四极杆飞行时间质谱对样品进行检测，并利用XCMS软件对原始数据进行归一化和峰值提取。结合主成分分析和正交偏最小二乘法判别分析进行代谢谱分析，成功筛选出9种上调物质和11种下调物质作为潜在生物标志物。研究结果显示，4-氯-3-甲基苯酚通过影响甘油磷脂、嘌呤等物质代谢及苯丙氨酸、酪氨酸等氨基酸的生物合成，对斑马鱼产生毒性作用。

相较原始的XCMS软件，XCMS Online得益于其统计分析的功能，在探究环境暴露对生物体毒性影响方面显得更有优势，用户只需针对处理后的结果进行代谢物分析与代谢通路分析，省去了许多手动分析步骤。张彦坤等^［30］^选取暴露于苯乙烯微塑料水体中的剑尾鱼为研究对象，利用Agilent 1290 Infinity LC与Triple-TOF 5600联用系统分析剑尾鱼的肝脏样品，使用XCMS Online平台进行质谱数据分析，以输出的潜在差异代谢物列表为基础，与Mass Bank的开放数据进行精确质量和二级质谱的匹配，对代谢物结构进行鉴定。最终筛选出与肝脏中的能量代谢、糖代谢、氨基酸代谢、炎症反应和氧化应激有关的8种代谢水平改变的差异代谢物，这些代谢物会干扰脂代谢、改变和神经毒性有关代谢物的表达。

## 3 研究展望

尽管XCMS软件在环境科学领域的应用正受到越来越多的关注，但该软件仍处于发展阶段，存在进一步改进和完善的潜力。在数据处理能力方面，XCMS在处理大规模数据时内存占用高、易崩溃，有案例显示当处理250个或更多的数据文件时，程序会反复崩溃^［31］^，这严重制约其在环境大样本研究中的应用。在分析准确性方面，XCMS在特征检测的过程中易将噪声误判为有效信号，导致假阳性率较高，影响分析结果^［32］^。环境样品中通常含有多种具有复杂的化学组成和结构类型的化合物^［33］^，XCMS在处理这些复杂数据时可能会出现错误或漏检情况，这会影响结果的准确性和可靠性^［34］^。另外，XCMS还存在用户交互界面差、自动化程度不高、聚类结果分离度不足、聚类与分离精度待提升、跨平台数据的可比性较为局限、参数优化与算法鲁棒性有待增强等问题。当前市面上质谱仪器类型众多，样品经处理后会产生不同的数据格式，鉴于XCMS软件只能处理特定的数据格式，这要求在将原始数据导入XCMS前必须进行格式转换，这无疑增加了数据处理的时间成本^［35］^。在未来研究中，XCMS可处理数据格式类型应作进一步拓展，以减少原始数据转化所需的时间。综上所述，XCMS作为一款新兴的质谱数据处理软件，在环境科学领域的污染物识别、代谢转化以及毒性效应分析等方面发挥着巨大的作用，同时还有诸多需要发展和完善的环节，仍需在算法鲁棒性、数据兼容性和数据库建设等方面进行系统性升级，未来随着这些技术瓶颈的突破，XCMS有望为环境科学研究提供更强大的支持。
